# Predictors of the highest long-term care expenditures in Japan

**DOI:** 10.1186/1472-6963-11-103

**Published:** 2011-05-17

**Authors:** Pedro Olivares-Tirado, Nanako Tamiya, Masayo Kashiwagi, Kimikazu Kashiwagi

**Affiliations:** 1 Department of Health Services Research, Graduate School of Comprehensive Human Sciences, University of Tsukuba, 1-1-1 Tenno-dai Tsukuba, Ibaraki, 305-8575 Japan; 2 School of Nursing, National College of Nursing,1-2-1 Umezono, Kiyose, Tokyo, 204-8575 Japan

## Abstract

**Background:**

In Japan, as the number of elderly covered by the Long-term Care Insurance (LTCI) system has increased, demand for long-term care services has increased substantially and consequently growing expenditures are threatening the sustainability of the system. Understanding the predictive factors associated with long-term care expenditures among the elderly would be useful in developing future strategies to ensure the sustainability of the system. We report a set of predictors of the highest long-term care expenditures in a cohort of elderly persons who received consecutive long-term care services during a year in a Japanese city.

**Methods:**

Data were obtained from databases of the LTC insurer of City A in Japan. Binary logistic regression was used to examine the predictors of the highest long-term care expenditures. We used a simplified model that focused on the effects of disability status and type of services used, while controlling for several relevant factors. Goodness of fit, a multicollinearity test, and logistic regression diagnostics were carried out for the final model.

**Results:**

The study cohort consisted of 862 current users of LTCI system in city A. After controlling for gender and income, age, increased utilization rate of benefits, decline in functional status, higher care needs level and institutional care were found to be associated with the highest LTCI expenditures. An increased utilization rate of benefits (OR = 24.2) was a strong main effect predictors of the high LTC expenditures. However, a significant interaction between institutional care and high care need level was found, providing evidence of the combined effect of the two covariates.

**Conclusions:**

Beyond to confirm that disability status of elderly persons is the main factor driving the demand of LTC services and consequently the expenditures, we showed that changes in utilization rate of benefits -a specific insurance factor- and the use of institutional care conditional on the high care level, were strongest predictors of the highest LTC expenditures. These findings could become crucial for tracking policies aimed at ensuring financial sustainability of LTCI from a public insurer perspective in Japan.

## Background

Face to the challenge of an aging population and its impact on social security system, it is important to improve our understanding of the factors associated with LTC expenditures among the elderly. From a public insurer's perspective, it is desirable to account for information concerning factors associated with higher levels of expenditures in the LTCI system. In this study, we attempt to quantify an association between a set of socio-demographic and insurance variables and LTC expenditures. We examined the likelihood of belonging to a high expenditure group in a cohort of elderly users of the LTCI system in a Japanese city, focusing on the influence of disability status and utilization of facility services. We anticipate the results of these analyses can help policy-makers address future long-term care policies that contribute to the financial sustainability of the LTCI system. This study will soon be complemented with the results of predictors of the lowest expenditures to complete the framework of LTCI expenditures in City A.

### Outline of Japanese Long-Term Care System

In the last decade, as aging populations have increased in developed countries, the social and economic consequences are becoming important public policy issues, particularly in the field of social security systems (i.e., pensions, health care, long-term care systems [1-3]). In Japan and most of developed countries, as the number of elderly people with disabilities or requiring support in their activities of daily living (ADLs) increase, the demand for long-term care (LTC) services has also increased and consequently, the expenditures of the LTC system are growing steadily, threatening the financial sustainability of the system [2-5].

In Japan, the percentage of people aged 65 or older was 12.1% in 1990 [6,7]. Since then, the Japanese population has aged rapidly. As of October 1, 2006, the total Japanese population reached 127.8 million and the proportion of elderly population was 20.8%, the highest in the world. According to estimates of the Japanese National Institute of Population and Social Security Research (NIPSSR), based on Census 2005, it is expected that the elderly, aged 65 or over, will increase annually by about 1 million people during the 2012-2014 period when the "baby-boom generation" (i.e., people born in 1947-1949) reaches 65 [6]. By the years 2025 and 2050, the elderly population is expected to reach 30.5% and 39.6% of the total Japanese population, respectively [6,7], and estimates projected by the OECD, depending on the scenarios, demographic effects, and cost-pressure or cost-containment, the projected LTC expenditures for Japan could reach 2.3%, 3.1%, and 2.4% of GDP by 2050, respectively [4].

To deal with the accelerated aging of Japanese society and the increased needs for nursing care for the elderly, in April 2000 the Japanese government introduced a Long-Term Care Insurance System for the elderly (hereafter "LTCI," i.e., *Kaigo Hoken*) [7-9]. The main purposes of this system were to promote independent living of the elderly in the community, to share the burden of caring for the elderly among all members of society, and to lessen the burden on family caregivers [7-12]. Evidence from micro-level household data suggested that the introduction of Kaigo Hoken helped Japanese households to reduce income losses associated with a disabled family member [13].

The LTCI system relies on a mandatory social insurance model, financed partially by general taxes (50%) and a designated insurance premium (50%). Universal benefit entitlement for the elderly are based strictly on the extent of physical or mental disability, regardless of means or whether any potential informal caregiver network is available. Since 2000, Kaigo Hoken explicitly are excluded any means-tested services and only services, not monetary benefits, are provided. Municipalities are LTCI insurers and administer LTCI based on national guidelines; each determines its own budget and insurance premiums for its residents. Premiums for elderly persons (Category 1) vary by income status of the insured and by municipality. Premiums for Category 2 insured persons (age range 40-64) are collected with health care insurance and pooled at the national level [8,9,11].

A national standardized process certifies an applicant's assistance/care needs level and defines the monthly limit of benefits. Since April 2006, there have been seven levels of certification under Kaigo Hoken; the two lightest levels are "assistance required" (*yo-shien*) and the remaining five levels refer to "care required" (*yo-kaigo*) [14,15]. Those certified in the *yo-shien *category can only use community care or preventive services, to help them to lead self-supporting lives while maintaining their present physical condition as long as possible [14].

The insured certified in *yo-kaigo *categories can receive home-based, community-based, or institutional care services. In Japan, only services available in LTCI system are delivered by providers. Services are delivered on a pay-as-you-go basis from municipal government-approved providers and facilities; most are private for-profit or non-profit firms [16]. Because the price for each service is set by the government and is the same in each region, providers compete for customers on the basis of their preferences and perceived quality of services [8,12,16,17].

Theoretically, users are free to choose services, but in reality, care-managers certified by Prefectures make care plans according to each applicant's certified care needs level, living environment, and requests from the user and family [8,14,15]. Then, a care plan is designed and the process concludes with a contract between a care provider firm and the user. However, beneficiaries are re-evaluated every 6 months and they may request changes to the care plan and, if dissatisfied, change the manager and/or provider. Under the LTC plan, when users receive LTC services, a fixed burden of 10% of the service cost is paid directly to the provider as a co-payment. However, Kaigo Hoken also considers subsidies, that refers to the benefits assumed by the Social Welfare to compensate poor people mainly in the former care needs level and it consists in the exemption of copayments[9].After enactment of the LTCI law (2005), for those living in a nursing care facility, housing expenses and meal fees were charged under the contract with the facility on top of the 10% co-payment [8,9,14].

The benefits provided by the Japanese LTC system after LTCI law enactment (April,2006) include services of care prevention benefits and services of long-term care benefits[9]. Services of care prevention benefits includes nursing care prevention services- designated and supervised by prefectures level- and nursing care prevention support -designated and supervised by municipality level. Additionally, services of long-term care benefits includes both at home and facilities services. The main categories of at-home care services include home-visit care, home-visit nursing, home-visit bathing service, home-visit rehabilitation, management guidance for in-home care and allowances for rental service of welfare equipments. In-home based services also include commuting services (e.g., day services) and short-stay daily life services. Commuting services in Japan define services delivered in community-based facilities where a user commutes to a day service center for the elderly and other facilities, where he/she is provided with personal care for bathing, toileting and eating, support for other daily-life activities, and physical exercises and return home the same day. In contrast, facilities for institutional care are divided into LTC welfare facilities for the elderly (special nursing homes), LTC health facilities for the elderly(geriatric intermediate care), and LTC medical facilities for the elderly(health care facilities for older adults). A user is admitted to a special nursing home for the elderly, where he/she s provided with personal care for bathing, toileting and eating, support for other daily-life activities, physical exercises, and assistance for health management and recuperation. On the other hand, 'geriatric intermediate care' and 'health care facilities for older adults', both defines LTC institutions where residents are under stable medical conditions but require rehabilitation or nursing or personal care. The main difference is that the former are licensed under LTCI law and the second are not included in the LTC program but offered under the national healthcare system. Other differences are professional staff composition, room sizes and fees [9].

According to data from MHLW, the disability status in the elderly population in Japan, considering the ratio of the total number of certified elderly for LTC support/care versus the total elderly population as a measure of disability prevalence, reached 9.9% at the time the LTCI system was implemented (April, 2000). By 2006, the disability prevalence rate had increased to 16.3%. [18,19]. On the other hand, examining the effect of the long-term care services on disability status of the elderly users of *Kaigo Hoken *in the period 2002-2007 in Japan, an annual average rate of improvement in disability conditions was 9% in the lowest care needs level (Care Level 1), 13% in the mid care needs level (Care Levels 2, 3) and 10% in high care needs level (Care Levels 4, 5). Additionally, the annual decline in disability status in the same period for those in the lowest care needs level (Support Level, Care Level 1) was 23%, in the mid care needs level(Care Levels 2, 3) was 24%, and 17% in Care Level 4 [20].

### Utilization rate of Benefits

The utilization rate of insurance benefits (hereafter, URB; i.e., the proportion of insurance benefits units effectively used by a recipient over the fixed limits of benefits defined by each certified care needs level in the Japanese LTCI system) depends on the care plan designed by the care manager and the family. Roughly, the average URB of the LTCI system for 2006 was 48.4% and by 2007, decreased to 47.2%. Certainly, the highest URBs are associated with the highest care needs level. In April 2006, the URBs of Care Levels 3, 4, and 5 were 50.3%, 53.5%, and 53.9%, respectively. Subjects in Care Level 1 exhibited the lowest URB (36.9%) [20]. According to official data of the All-Japan Federation of National Health Insurance Organizations (*Kokuho Chuokai*, 2010), a calculated composition of spending by care needs level for FY2007 showed an exponential increase in annual average expenditures, from ¥37,083 of Support Level 1 to ¥443,202 of Care level 5 [21].

### Long-Term Care Expenditures in Japan

In Japan, as the LTCI system became established, certified users of LTCI have increased rapidly, and the demand for LTCI services has experienced a remarkable expansion and, consequently, the expenditures of the LTCI are growing dramatically, threatening the financial sustainability of the system [9,22]. The total LTCI users in 2000 were 6.8% of the total elderly population, increasing to 13.1% by 2006. The total LTC expenditure for FY2006 was ¥ 6.36 trillion (US$ 54.7 billion) representing 1.2% of GDP and a growth of 100% in the LTC budget since 2000 [8,9,23]. A revised LTCI law, enacted in June 2005, sought to ensure the sustainability of the system by establishing, among other measures, a prevention-oriented system, review of the facility benefits, and a review of financial burden and system management [9].

Research on the demand for LTC services, as well as research on LTC expenditures, is usually based on aggregated and/or historical data about LTC expenditures and having countries as the unit of analysis, and is used to identify economic, demographic, and institutional determinants of futures expenditures. Econometric macro- and micro-simulation models are the most common methods used to investigate future trends in demand and expenditures of long-term care, based on aggregate data and demographic projections [24-26]. In the last decade in Japan, many studies have estimated the future impacts of the aging population on social security systems. Most of this studies conducted at macro-level, assist in reducing uncertainty regarding the extent of the demand for health and long-term care services and their associated costs and expenditures for elderly people [4,10,16,17,27,28].

Beyond the official information communicated periodically by the Ministry of Health, Labour, and Welfare (hereafter, MHLW) and other organizations of the Japanese government, few empirical studies on Japan's LTC expenditures are available in English. Karlsson et al. [29] argued for a considerable increase in LTC spending and a growth in the relative burden of LTC, because the working population is estimated to decline and LTC costs will increase, given an increase in the demand for formal care, because in Japan informal care is not reimbursed. Tsutsui et al. [8] argued costs would skyrocket in Japanese LTCI before the amendment to the law in June 2005, due to removal of means-tests for services, an increased demand for institutional services and municipalities minimal control over the quantity and type of services provided, suggesting a supplied-induced demand. However, Shimizutani [30] and Noguchi [31] found little evidence that a higher number of providers stimulated higher monthly expenditures for care services.

Ikegami [32] argued that beyond population aging, demand for LTC services increased as a result of an increasing number of certified eligible people becoming aware of their entitlement, and the concurrent expanding supply of service providers. In the same paper, Ikegami [32] suggested that an excess of demand for institutional care, specifically for "housing" (i.e., group homes for less severe dementia patients) and "special facilities" (e.g. nursing homes not owned by social welfare organizations and "assisted living"-type housing), had increased from 1% to 6% of total LTC expenditures in the period from 2000 to 2005. Ogura et al. [33] suggested that most of the growth in the expenditures for LTC arose from the home-care sector, essentially due to the increase in the number of certified elderly and the expansion of the service supply.

While disability status and age/longevity are undoubtedly driving forces in long-term care expenditures, a more subtle concern pertains to the competing roles of LTC insurance utilization and type of services used by LTC beneficiaries. Numerous studies document that disability status, rather than age *per se*, plays a pivotal role in long-term care expenditure predictions [5,34-37]. On the other hand, in Japan a little evidence exists regarding how insurance benefits utilization or the type of long-tem care services affects LTC expenditures. In particular, Campbell et al. [12] compared the composition of per-capita LTC spending by the elderly in USA, Japan, and Germany in 2005, and showed that, in Japan, 60.6% of total LTC expenditures were by the elderly in institutional care [12].

In summary, concerning LTC expenditures in Japan has been argued that the growing LTCI expenditures can be attributed to a steady increase in the demand of in-home care services due to the increase of certified number of elderly in lower care needs level[30,32] and an increased demand for institutional care explained by the universal entitlement of the LTCI system that permit access to the care to all elderly according to their needs regardless of the economical or social conditions [8,15]. On the other hand, as LTCI system in Japan is based on an open provider's market, a supplier-induced demand (SID) effect has been examined by some authors, concluding that expansion of long-term care expenditures has not been caused by supply-side factors[30,31].

## Methods

### Data

This study used a retrospective design based on individual-level data obtained from the database of the LTC Public Insurer of City A in Japan. Consent for use of the data was approved by the municipal government of City A after a formal application and explicit pledge to randomize all data and remove any individual identifiers to protect the privacy of the personal data supplied. Ethical considerations were examined in accordance with Japanese epidemiological guidelines for secondary data analysis. Ethics approval was obtained from the University of Tsukuba Ethical Committee, Japan.

The dataset comprises two registers: the LTCI benefits register and household income levels for the LTCI system in City A. The former contains monthly information from the provider's claims for reimbursement and reflects LTCI recipient data, services provision, and associated expenditures. It includes the register code, date of birth, gender, care needs level, date of provision, type and amount of services used, insurance benefits used, expenditures, co-payments, and subsidies. The household income level register for the LTCI system contains the register code and a classification code of the household income level of the current users of LTCI services, based on household members' taxation and taxable pension income of the elderly to estimate an LTCI premium amount. These two registers were linked using the register code as the key linkage. The combined dataset thus comprised basic demographic characteristics and the long-term care history of all individuals who received benefits from the LTCI system in City A.

### Study population

City A is located in a suburban area approximately 100 km West of Tokyo. The estimated population as of October 1, 2006 was 52,343 and the proportion of elderly persons (aged 65 or over) was 20.8% [38]. This proportion is the same as the average in Japan [39].

The target group of this study comprised all elderly persons certified for LTCI who received long-term care services during 12 consecutive months, from July 2006 to June 2007 in City A (*n *= 885). Because we focused on predictors of the highest LTC expenditures, we decided also to exclude individuals who exhibited a marginal utilization of LTC insurance benefits (*n *= 23), to improve the stability of the model. Operationally a marginal utilization of LTC insurance benefits (URB) was defined as an URB less than 10% at baseline time or at the end month of the study period.

In July 2006, a total of 1,197 elderly persons used LTCI services in city A. Of them, 862 persons were eligible for this study, representing 72% of the total elderly users of the LTCI system in City A.

### Data analysis

The total expenditure for each subject in the study population was calculated as the sum of the total monthly expenditures claimed by providers during the study period. The data on the total expenditures of the sample was sorted by values, ordered from largest to smallest. Then, using a quartile function, we identified the top 25% of individual total expenditures group in the sample population. The cut-off (Q3) to choose the target group was ¥ 3,029,500 and participants in the top 25% of the total expenditures group were considered as the high expenditure subgroup. This target subgroup represented 45% of the total annual expenditures of the study population.

For each individual in the sample, a monthly URB was calculated as the proportion of insurance benefits units used by a recipient over the fixed limits of benefits defined by each certified CNL in the Japanese LTCI system (see Table 1).

**Table 1:** Benefits limits standard amount for in-home services.

Level of long-term care need	Benefit limit standard amounts
Requiring Support 1	**4,970 **units/month
Requiring Support 2	**10,400 **units/month
Requiring Long-term Care 1	**16,580 **units/month
Requiring Long-term Care 2	**19,480 **units/month
Requiring Long-term Care 3	**26,750 **units/month
Requiring Long-term Care 4	**30,600 **units/month
Requiring Long-term Care 5	**35,830 **units/month

1 unit = ¥ 10 to ¥ 11.05 (subject to region and kinds of service) Source: Annual Health, Labour and Welfare Report 2008-2009 MHLW.Japan

### Conceptual model

From a theoretical point of view, and following the simple approach proposed by Norton [40], we assume LTCI expenditures for an individual as a function of a number of factors, including socio-demographic characteristics (age, gender, education, marital status, family structure), economic circumstances (income, insurance coverage), disability status (based on standard assessments of dependency and care needs), and geographic factors (provider supply and regional services utilization patterns). Rather than estimate a fully specified model, we used a simplified model that focused on the effects of the utilization rate of insurance benefits, disability status and institutional care services used, while controlling for a limited number of relevant factors.

### Dependent variable

As with health care expenditures, long-term care expenditures have a skewed, rather than a normal, distribution and a log transformation can be used for the OLS estimation (Manning & Mullahy [41]). However, we were not interested in predicting log expenditures; rather, our interest was to identify which factor(s) predict membership in the high expenditure group in City A. A dichotomous variable indicating membership in the high expenditure group was defined for each participant as the outcome variable (Y = 1). Analysis of this dependent variable was then conducted from the perspective of the LTC insurer.

### Independent variables

Research on LTCI expenditures does not offer a specific conceptual framework at the individual level to guide the selection of independent variables, nor does it suggest how the variables may interact to influence LTCI expenditures. On the other hand, despite that have been desirable to include information about the effect of the change in Japan's family structure in favor of nuclear family or the increase in female labor force participation rate in our model, unfortunately data about this factors is not available. Data about informal care is not captured at insurer level in Japanese system because, universal benefits entitlement for the elderly are based strictly on the extent of physical or mental disability, regardless of economical conditions or whether potential informal caregiver network are available. However, based on empirical evidence available in the literature, we assumed that an individual's probability of incurring high LTCI expenditures was affected by age, individual disability status, income level, insurance coverage, and consumption pattern of services, as the main expenditure drivers.

There is evidence that LTCI expenditures depend on the age of the elderly [10,23]. Age was included as a categorical variable with four levels: less than or equal to 74 years (reference group), 75-84 years, 85-94 years, and equal to or greater than 95 years.

A univariate analysis was carried out and gender and income variables were not significant, but were forced to remain in the model for adjustment. Gender was a dichotomous covariate, where female was chosen as a target group. The household income levels for the LTCI system in City A were classified in six levels, from lowest to highest. We designed a categorical variable with three levels, aggregating the two lower, the two middle, and the two highest levels. The lower category was included in the model as a reference group.

A relative change in URB was calculated for each subject in the study as a measure of the change in insurance coverage during the study period. It was calculated as the proportion of the difference between URB at the end of the study period and the URB at the baseline time over the URB at the baseline time. A dichotomous covariate was designed; an increase in the relative URB was our variable of interest; otherwise, no change or a decrease in the relative utilization rate was set as the reference group.

The disability status of the participants, one of the main areas of focus in our study, was included, with two dimensions being considered: a static dimension, represented by the Care Needs Level at the end of study period, and a dynamic dimension, capturing the change in the disability status during the study period. The Care Needs Level contains seven categories: two support levels - Support Level 1 and 2, and five care categories - from Care Level 1 to 5, lowest to highest, respectively. Additionally, the Care Needs Level was included as a categorical variable with three categories: a lowest category including both support levels and Care Level 1, a mid level category formed by Care Level 2 and 3 and finally, a highest category, including Care Level 4 and 5. The lowest category was chosen as the reference group.

Change in disability status was calculated by subtracting the baseline Care Needs Level from the Care Needs Level at the end of the study period. If a participant change in Care Needs Level was calculated to be greater than zero or equal to zero when the subject remained at Care Level 5 throughout all study periods, this was defined as a decline in dependency level was defined and coded as decline in functional status. Otherwise, participants whose change in Care Needs Level was equal to or less than zero at all needs levels other than Level 5 were defined as unchanged or improve of the disability status and coded as unchanged functional status. A dichotomous covariate having a decline in functional status as the variable of interest was designed.

A breakdown by type of services of the total LTCI expenditures in Japan for FY2006 shows a significant proportion spent on facility services (45%), followed by home-based services (36.5%) and commuting services (18.5%) [20]. The per-capita average expenditures by type of facility services in April, 2006 was ¥253,000 in a special nursing home, ¥262,000 in geriatric intermediate care, and ¥373,000 in health care facilities for older adults [20]. In our data, at the end of the study period, the main consumption patterns of services included facility services (32%), commuting services (26%), in-home services (8%), and mixed services (i.e., more than one of the categories mentioned previously, excluding facilities) (30%). Because evidence cited earlier [20,23] showed that monthly average expenditures by the elderly for facility services (institutional care) grew steadily in Japan, we focused on this aspect. A dichotomous covariate was designed and facility services utilization was chosen as the variable of interest. Utilization of any other LTCI service was considered as the reference group.

### Statistical analysis

A descriptive analysis was undertaken to understand the relationship between the high expenditures group and the covariates set. The chi-squared test was used to analyze the relationship between the outcome variable and covariates set (Table 2).

**Table 2:** Descriptive characteristics of the study population (n:862)

	Long-term Care Expenditures		
Covariates	higher (n: 216)	non-higher (n: 646)	Total
	
	n (%)	n (%)	n (%)
Age*			
**< = 74 y-old (ref)**	**20 **(9.3%)	**103 **(15.9%)	**123 **(14.3%)
**75 -84 y-old**	**84 **(38.9%)	**247 **(38.2%)	**331 **(38.4%)
**85-94 y-old**	**81 **(37.5%)	**263 **(40.7%)	**344 **(39.9%)
**> = 95 y-old**	**31 **(14.4%)	**33 **(5.1%)	**64 **(7.4%)

**Gender**			
**male (ref)**	**50 **(23.1%)	**184 **(28.5%)	**234 **(27.1%)
**female**	**166 **(76.9%)	**462 **(71.5%)	**628 **(72.9%)

**Income level**			
**low (ref)**	**29 **(13.4%)	**90 **(13.9%)	**119 **(13.8%)
**middle**	**163 **(75.5%)	**476 **(73.7%)	**639 **(74.1%)
**high**	**24 **(11.1%)	**80 **(12.4%)	**104 **(12.1%)

U Rate Benefits(URB)*			
**equal or decrease(ref)**	**166 **(76.9%)	**587 **(90.9%)	**753 **(87.4%)
**increase**	**50 **(23.1%)	**59 **(9.1%)	**109 **(12.6%)

Change in functional status*			
**unchanged(ref)**	**108 **(50.0%)	**441 **(68.3%)	**549 **(63.7%)
**decline**	**108 **(50.0%)	**205 **(31.7%)	**313 **(36.3%)

Care needs level *			
**low care need level (ref)**	**3 **(1.4%)	**285 **(44.1%)	**288 **(33.4%)
**middle care need level**	**48 **(22.2%)	**276 **(42.7%)	**324 **(37.6%)
**high care need level**	**165 **(76.4%)	**85 **(13.2%)	**250 **(29.0%)

Type of services*			
**others(ref)**	**41 **(19.0%)	**543 **(84.1%)	**584 **(67.7%)
**facilities**	**175 **(81.0%)	**103 **(15.9%)	**278 **(32.3%)

Test for statistical differences between high expenditures and non-high expenditures groups were conducted using X^2 ^test. * p < 0.0001

A binary logistic regression model was used to examine the effect of the covariates on total expenditures in the high expenditures group. The modeling proceeded in three stages. First, variables the chi-squared test for which had a *p- *value < 0.25 (Hosmer-Lemeshow [42]) in a univariate analysis were selected to be included in a preliminary model. Second, including all set of covariates to identify variables which make contribution to the model in presence of other variables we build a main effect model. A stepwise procedure was useful to identify the relative importance of the covariates set in the model. The inclusion and exclusion criteria for the stepwise regression were both 15% (Hosmer-Lemeshow [42]). The Wald statistic test for each covariate was examined, and those with a significant level p < .05 were included in the final model. These variables were: a) age, b) increase in relative URB, c) decline in functional status, d) Care Needs Level, and e) utilization of facility services. Gender and income were not statistical significant but forced to stay in the model to controlling for their effects. Finally, the interaction among some explanatory variables was examined. A second-order interaction between facility services utilization and the highest care needs level category was significant and included in the final model.

Multicollinearity was examined via a correlation matrix and multicollinearity diagnostic statistics, from a regression of the covariates set on an "exogenous" variable i.e., a log-transformation of total expenditures. A logistic regression diagnostic was carried out to identify influence or outlier covariate patterns (Hosmer-Lemeshow [42]). Plots of the change in Pearson *χ^2 ^*and Deviance *χ^2 ^*against predictive probability was used to detect outliers or influential points. One case was identified and excluded from the analysis, improving the overall goodness-of-fit of the final model. Hosmer-Lemeshow and -2 log likelihood tests were used to check goodness-of-fit of the final model.

The results are reported as odds ratios and differences in predicted probabilities of high expenditures, conditional on the vector of predictor variables. For each dichotomous or categorical variable, the odds ratio indicated the ratio of the odds of belonging to the higher expenditures group for the given category, relative to the reference group, while controlling for other covariates. The method of logit differences was used to estimate the odds ratio for the interaction term; the confidence intervals for the odds ratio were calculated using standard error methods (Hosmer-Lemeshow [43]).

To estimate the overall change a given covariate had on the outcome variable in terms of the differences in the predicted probabilities between target and reference group, we use the delta-p statistic, according to the method suggested by Cruce [44]. The delta-p statistic is a measure of a discrete change in the estimated probability of the occurrence of an outcome, given a one-unit change in the independent variable of interest, with all other variables held constant at their mean values. Delta-p is calculated as the difference in the probability of the occurrence of an outcome between a target and reference group (Cruce [44]). However, following recommendations by St. John (1991) [45] the use of the delta-p statistic was limited only to those covariates found to be significant in the model, because there is no known procedure to estimate the statistical significance of delta-p (Cabrera [46]). All analyses were conducted using the SAS software, version 9.1 for Windows(SAS Institute Inc.).

## Results

### Descriptive analysis

The characteristics of the sample population are summarized in Table 2. The sample comprised 862 individuals with a mean age of 83 years (standard deviation, SD = 7.7) and 73% were females. Most of the subjects (74%) belonged to the mid-income level, 14% were in the low level and 12% in the high income level.

Thirteen percent of subjects showed an increase in the relative URB during the study period. A decline in functional status was observed in 36% of the sample at the end of the study period. The care needs level distribution in the sample was; 33% at low care needs level, 38% at mid care needs level and 29% at high care needs level. The breakdown of subjects using facility services at the end of study period was as follows: 32% of the total number of subjects reside in facility services; 54.7% of these facility users belonged to the high care needs level; and 61% of subjects certified at high care needs level used facilities services at the end of study period.

The high expenditures group comprised 216 subjects with a mean age of 85 years (SD = 7.8). Most were females (77%) and the annual average expenditure was ¥ 3.4 million per person (min. = 3.0 million, max. = 5.0 million). The higher expenditures group represents the 45% of the total annual expenditures of the study population.

### Model goodness of fit Statistics

The final model on the highest LTC expenditures in City A, it was expressed as follows:

***Logit (HIGHEXPij) ***= *β0 + β1GENDERij + β2AGEdummiesij + β3INCOMEdummiesij +*

β4URATEij + β5FUNCTij + β6CARELEVdummiesij + β7 TYPESSij+

β8(FACILITYSSij ×HIGHCARELEVij) +

β9(FACILITYSSij ×MIDDLECARELEVij)

Overall, goodness-of-fit suggested that the model was significant and adequate. The test for overall fit of the model indicated that the model with selected covariates (-2 log L = 364.831) was superior to the model with interceptor only (-2 log L = 967.791). The Hosmer-Lemeshow test result was 0.441, indicating that the model predicts the data well. Estimates of pseudo R^2 ^in our model shows a Cox & Snell R^2 ^= 0.505 and a Nagelkerke R^2 ^= 0.747 states that the model manages to explain over 50% of the variance of the dependent variable and indicated an acceptable model fit. The overall logistic regression model was highly significant at the 5% level, as indicated by the likelihood ratio. Wald and Score tests (p < 0.001) of the global null hypothesis suggests that a specific coefficient of the covariates equals zero, then at least one coefficient (*β) *in the model is non-zero.

Multicollinearity was examined using a correlation matrix and diagnostic statistics. A moderate expected association between some categorical variables (age and income) was observed. The Variance Inflation Factor (VIF) for each variable was also examined. Values of VIF ranged from 1.06 to 2.36, indicating the non-existence of multicollinearity in the model.

Values for indices of rank correlation indicated that the predictive ability of the model was adequate; 96% of the pairs were concordant. Values of Sommer's D, Gamma and C statistics were sufficient (> 0.92). A large percent estimated area (96.2%) under the receiver operating characteristic (ROC) curve indicated adequate fit of the model. For the probability of event = 0.52 the sensitivity (79%) and specificity (95%) of the model were sufficient.

### Logit results

Estimates for the parameters obtained through the maximum likelihood estimation method with 95% Wald's confidence limits for the final model are shown in Table 3.

**Table 3:** Estimated coefficients, Standard errors, p-values and 95% Confidences Intervals for the final logistic regression model for high expenditures in city A (n:861)

parameters	coeff	**S.Err**.	Wald *X^2^*	*p*-value	C.I. (95%)
**female**	0.418	0.3387	1.52	0.217	(-0.246, 1.082)
**75-84 y-old**	1.666	0.4995	11.13	**0.001**	(0.687, 2.645)
**85-94 y-old**	1.340	0.4966	7.28	**0.007**	(0.367, 2.313)
**= > 95 y-old**	1.567	0.6179	6.43	**0.011**	(0.356, 2.778)
**middle income**	-0.452	0.4059	1.24	0.266	(-1.248, 0.344)
**high income**	-0.156	0.5564	0.08	0.780	(-1.246, 0.935)
**decline in functional status**	0.703	0.3146	4.99	**0.026**	(0.086, 1.320)
**increase URB†**	3.187	0.4702	45.93	**< .0001**	(2.265, 4.108)
**facility services use**	1.044	0.1673	38.92	**< .0001**	(0.716, 1.372)
**middle care needs level**	1.592	0.3363	22.39	**< .0001**	(0.932, 2.250)
**high care needs level**	3.624	0.3864	87.96	**< .0001**	(2.867, 4.382)
facility ss *middle care needs level	0.400	0.3296	1.48	0.225	(-0.246, 1.046)
facility ss * high care needs level	1.414	0.3461	16.69	**< .0001**	(0.736, 2.093)
**constant**	-2.765	0.6524	17.96	**< .0001**	(-4.044, -1.486)

**URB†**: Utilization Rate Insurances Benefits

The logit results indicated that after controlling for gender and income levels, covariates such as age, increased URB and decline in functional status significantly affected the probability of high expenditures in the LTCI system in City A. However, an interaction between use of facility services and high care needs level was significant, providing evidence for the combined effect of the two covariates.

The adjusted OR and delta-p statistics for the final model of high expenditures in City A are shown in Table 4. Controlling for other variables in the model, it was found that to belong to mid or high care needs level were strongest predictors in our model. The odds of being in the high LTC expenditures group are about 24 times greater for subjects in a mid care needs level as they are for the lowest care needs level. The effect of higher care needs level was involved in an interaction term with facility services utilization in our model. Our analysis also showed that an increase in relative URB was a strong predictor of high LTC expenditure. The odds of being in the high LTC expenditures group when URB increase was 23.5 times higher than those whose URB remain unchanged or decreased during the study period.

**Table 4:** Estimated adjusted Odds ratio, 95% Confidences intervals for odds ratio, and delta-p statistics for the final logistic regression model for high expenditures in city A (n:861).

covariates	odds ratio	95% CI	*delta-p*
**main effects**			
**male**	1.00		
**female**	**1.53**	(0.80, 3.02)	**-**
			
**< = 74 y-old**	1.00		
**75 -84 y-old**	**5.23**	(2.03, 14.45)	**0.211**
**85-94 y-old**	**3.75**	(1.46, 10,31)	**0.151**
**> = 95 y-old**	**4.71**	(1.44, 16.15)	**0.193**
			
**low income**	1.00		
**middle income**	**0.63**	(0.29, 1.41)	**-**
**high income**	**0.85**	(0.28, 2.49)	**-**
			
**equal or decrease URB†**	1.00		
**increase URB†**	**23.53**	(9.62, 63.99)	**0.575**
			
**unchanged functional status**	1.00		
**decline in functional status**	**2.02**	(1.10, 3.77)	**0.137**
			
**low care needs level**	1.00		
**middle care needs level**	**24.10**	(7.85, 106.77)	**0.426**
			
**interaction effect**			
high care needs level * facility			
**use facility services**	**105.60**	(41.5, 268.7)	**0.654**
**use others services**	**37.04**	(17.6, 77.9)	**0.325**

**URB†:**Utilization Rate Insurances Benefits

Moreover in Table 4 we can see a moderate impact of age and decline in functional status on high LTC expenditures. Controlling for other variables in the model, the odds of being in the high LTC expenditures group when subjects belonged to the 75-84 year age group were about 5.2 times higher than people aged 74 years or younger. Similarly, the odds of being in the high LTC expenditures group for subjects aged 85-94 years and over 95 years were 3.8 and 4.7 times higher than people aged 74 years or younger, respectively. Those classified in a decline in functional status had predicted odds of high LTC expenditures 2-fold higher than persons considered to be unchanged functional status.

Regarding the interaction term between facility services utilization conditioned by higher care needs level, as the coefficients of the two variables move in the same direction we estimated that for subjects using facility services, compared with those using other LTC services, when they are certified in the higher care needs level there is a 3-fold effect on high expenditures. On the other hand, when subjects that use facility services compared with those use another LTC services and they are certified in middle care needs level, the effect on high expenditures was 1.5 times higher, but this difference was not statistically significant (data no shown).

Also, we presents the result of the logistic regression model in terms of calculated delta-p statistics for significant covariates, according the method suggested by Cruce[44] because, interpreting a difference in predicted probabilities requires no specialized knowledge or advanced statistical skills. Subjects that exhibited an increase in relative URB had an estimated probability of belonging to the high expenditure group that is 57.5% higher than for those having an unchanged or decreased relative URB. Those, in the mid care needs level had an increased probability of belonging to the high expenditure group by 42.6% over those in the low care needs level.

A slight difference in the predicted probability of belonging to the high expenditures group was observed for age categories and decline in functional status. The estimated probabilities for being in the high expenditures group for subjects aged 75-84 years, 85-94 years and ≥95 years were 21.1%, 15.1%, and 19.3% higher, respectively, than the probability for subjects in the reference group (aged ≤74 years). Participants categorized with a decline in functional status had an estimated probability of being in the high expenditure group of only 13.7% higher than for those who had an unchanged functional status. This small difference could be explained by an insignificant difference between the patterns of services used by individuals in both categories.

The effect of facility services utilization conditional on the high care needs level, postulated in our model in terms of the difference in predicted probabilities of belonging to the high expenditures group was 65.4% higher than for those subjects who used other services and did not belong to the high care needs level. On the other hand, the difference in the estimated probability to belong to high expenditures group between who are residents on facilities and users of other LTC services was 33% when subjects are ranked as high care needs level.

In this model we postulate a specific hypothesis that involves the effect of an interaction term between facility services utilization and care needs level. Thus, in terms of the difference in predicted probabilities to belong at high expenditures group, the subjects on the high care need levels have been a probability 65.7% higher than the probability for those subjects use another LTC services and they not belong to high care needs level. On the other hand, the difference in the estimated probability to belong to high expenditures group between facility services users and other LTC services users is 35.2% when subjects belong to high care needs level. Given the non-linearity of the logit model, the interpretation of the coefficient of an interaction term lacks an intuitive interpretation. This conditional effect in a logit model is directly assessed through the "main effect" and its "interactive effect," given by the interaction coefficient [47]. In our model this effect is equal to the difference in predicted probabilities when the high care needs level variable increases from zero to one at different observed values of the facility services utilization covariate. This effect corresponds to the delta-p for the interaction term, which was 0.321 in the model. One interpretation is that the difference in the effect of care needs level by type of services on the estimated probability of belong to high expenditures group is 32%.

## Discussion

As the LTCI system has become established in Japanese society and the LTC supply has expanded, demand for LTC services has increased greatly and consequently the expenditures of the system are growing dramatically [9,22]. In Japan, the total LTCI expenditure for FY2007 was ¥6.9 trillion (US$ 58.5 billion) representing 1.3% of GDP and a growth of 8.9% over the FY2006 budget [18]. In response to this, and beyond set prices and types of LTC services delivered, the Japanese government has instituted important changes, contained in a 2005 LTCI Law amendment to ensure the sustainability of the system.

Most of the literature dealing with LTCI expenditures in Japan is at a macro-level and provides insight into complex and sensitive issues, such as future demand, costs, and financing alternatives in a country context [4,10,16,17,27,28,48]. However, at the micro-level, namely, the LTC insurer or individual level, there have been few empirical studies concerning LTCI expenditures in Japan and the available studies have focused primarily on aggregate approaches related to supply/demand factors [8,10,15,30,32].

This study set out to investigate factors associated with the higher LTC expenditures in an elderly cohort from a Japanese city. Our results demonstrated that an increase in relative utilization rate of insurances benefits as main effect was a strong predictor of the higher LTC expenditures. However, an interaction between institutional care utilization and higher care needs level, also was a significant findings in our study. Finally, although only slight, our logistic regression model picked up a positive effects of age and decline in functional status on high LTC expenditures. These findings can be used in future studies to understand expenditures trends from LTCI system by targeting the high risk groups that have been identified.

In the LTCI system in Japan, on average, recipients use only 48% (2006) of their benefits entitlement (range, 37-54%), but this is steadily increasing [12,20]. Certainly, the supervision by local government of the care manager's role is a crucial regulatory mechanism in this issue. Our results suggest that an increase in the relative URB is a strong predictor of high LTC expenditures. So we are probably noticing that utilization rate of benefits as an insurance variable, becoming a relevant factor involved in higher LTC expenditures. Then, under an insurer perspective the monitoring of URB, could be considered as a reliable indicator of the care managers performance on LTC expenditures.

In the long-term care field, there is consensus that disability of elderly persons is the main factor driving the demand for LTC services, for community-based or institutional services. Obviously, care needs level of elderly per se it is not a direct factor associated with high LTC expenditures, but it is a strong predictor of the demand of LTC services and consequently an important factor to explain LTC expenditures. Our model was able to demonstrate a significant interaction effect between facilities services utilization and higher care needs level -as a static measure of the disability status - providing visualization of the combined effect of these two covariates. These findings could be explained by the increased demand for nursing homes and intermediate geriatric facilities observed in the LTCI system in Japan, even after enactment of the law in 2005 that reduced economic incentives for institutionalization [8]. The annual demand for nursing home facilities in Japan has been increasing at a rate of 2-3% per year since 2006 [12,20] due mainly to longer waiting lists for institutional care at the present.

Although not the primary focus of this paper, a moderate impact of age and decline in functional status on high LTC expenditures were observed in our study. In spite, earlier studies are well documented that disability status - rather than age *per se*- plays a pivotal role in long-term care expenditure predictions [5,34-37], it is interesting to notice that age, although slightly, appears to be a significant factor in our model, the age effect was only significant in female gender, thus we recommend precaution with the interpretation of this result. On the other hand, the effect of a dynamic dimension of the disability status, even slightly, became an additional and significant predictor for higher LTC expenditures. The positive association between higher LTC expenditures and a dynamic disability measure in the adjusted model could be explained by the fact that a decline in functional status over the study period, determines changes in consumption pattern of services due to increased frequency or change of the type of services which, in turn, cause an increase of the LTC expenditures. Thus, this finding address to a challenging economical issue as it is the evaluation of the cost-effectiveness of LTC services in Japan.

Gender differences and household incomes were not statistically significant in both univariate analysis(p < 0.25) and multivariate analysis in our model, probably as a result of the 'welfare" structure of LTCI system in Japan, due to the eligibility for benefits is based solely on need and does not take into account the financial position or family structure of the users. However, as difference by gender in Japan shows one of the highest gender survival gaps in the developed world (women outlive men by 7 years (2009)) a separate analysis by gender it was conducted and a two-way interaction between gender and age covariates it was also investigated. In the men strata model, age as continuous or categorical variable was not statistically significant associated with the high expenditures group. Whereas among women, age as a continuous variable or as the three age categories, are statistically significant associated with the high expenditures group in a multivariate analysis (data not shown). The two-way interaction (gender*age) was not statistically significant in our model (data not shown). In spite of most of the men falling into youngest age categories in the target group, this effect it is not significant in a multivariate logistic model. Then, gender and household income were forced to stay in the model for controlling for their effects.

Despite a well documented literature on the importance of the role of the informal care provision in LTC systems and his economical effect on LTC expenditures, it should be notice that Japanese LTCI system -based on universal entitlements - does not consider any payment mechanism for family caregivers. Then, through lack of an informal care market is not possible to estimate informal care expenditures. In this context, just it is possible to estimates the opportunity-cost of time of the informal caregivers, but this issue is beyond the goal of this study. Furthermore, a recent study, based on data from a longitudinal survey of a nationally representative sample of the population over age 65 years in Japan, conclude that there is a substitution effect between formal and informal care but this effect vary by the characteristics of the informal caregiver. Thus, unmarried children -mainly daughter - and presence of children with a lower opportunity cost of time are more likely to provide care. Moreover, the results shows consistency with studies suggesting that actual of daughters-in-law, as the primary source of informal care under the traditional social norm, becoming less important in providing care than that of unmarried children[49].

Certainly, due to the nature of the data sources used, our analysis has several limitations. One weakness of our study and a possible source of information bias is that the data contain no information about supply factors (e.g., profit or ownership status of the providers, geographical density of providers or quality of services). Another possible source of information bias is that the data do not contain details about the potential influence of informal caregivers on expenditures. Another potential weakness is that our logistic regression model was not designed to control for possible endogeneity bias (i.e., an independent variable is correlated with the error term or an unobserved factor).

Finally, Fukawa [10], using a micro-simulation model based on physical disability, rather than age, concluded that estimates of LTC expenditures for the elderly in Japan will increase rapidly, rising to 3-4% of GPD by 2050. Concerning the sustainability of the LTC system, this author suggested that the only positive way to contain the expansion of LTC expenditures was to prevent the elderly from becoming dependent. Additionally, we demonstrated that from a public insurer perspective, the disability level is not the sole factor that must be taken into account.

Other factors, such as an increase in the relative URB and the types of services delivered, primarily related to institutional care, contribute significantly to explaining the high expenditures in our study. Their potential impact in determining future trends in LTC expenditures in Japan should be considered in future models.

## Conclusions

Beyond confirming that the disability status of elderly persons, measured as care needs level, is the main factor driving the demand of LTC services and consequently of the LTC expenditures, we demonstrated that others factors, such as changes in URB and the use of institutional care conditional on a high care needs level, were the strongest predictors of the highest LTC expenditures. Undoubtedly, these findings offer a new perspective in dealing with the challenge of retaining the sustainability of the LTCI system.

From the point of view of the LTC insurer in Japan (i.e., municipalities) the utilization rate of the insurance benefits could be a relevant indicator of the disability profile of the population and consequently of demand for LTC services. The significance of the increased URB as a main effect factor in our model could be interpreted as the impact of an increased demand of more complex LTC services by elderly users during the study period. On the other hand, the effect of decline in functional status -a dynamic measure of disability in our model- on LTC expenditures could be interpreted also as a change in the consumption pattern of services by the users during the study period. Thus, the ability to evaluate the quality and cost-effectiveness of the LTC services becomes a major challenge for insurers, managers, and providers of *Kaigo Hoken*. It is difficult to gain sufficient efficiency in the LTCI system without a clear knowledge about the effectiveness of the services delivered.

On the other hand, despite literature reports that institutional care has a pivotal role in long-term care expenditures, we demonstrated that institutional care interacting with higher care needs level is a relevant factor in explaining the highest LTC expenditures in our model. The association between institutional care utilization and higher care needs level or, in other words, the utilization of facility services by elderly with severe disability status, is a critical set of conditions related to LTCI expenditures in City A. The importance of these findings supports the need for a critical evaluation of the role of facilities services in the LTCI system. Some obvious questions include whether, these institutions should be considered as a final residence for the severely disabled elderly?, how much do the facilities services contribute to improving the disability status of their elderly residents?, and do the economic incentives in the institutional care sub-system operate to retain their users, or to reintegrate them as soon as possible into the community? This would seem to be a crucial issue for policymakers to examine the scope of these questions when the projected expenditures of the LTCI system threaten the sustainability of this system in Japan.

## Competing interests

The authors declare that they have no competing interests.

## Authors' contributions

PO-T carried out structuring the study design, statistical analysis, interpreting the data, and drafting the manuscript. NT supervised all the process as the corresponding author: participated in the design of the study, acquiring the data, interpretation of the data, and helped to finalize the manuscript.MK participated in designing this study, acquiring the data, and structuring the data set. KK helped to create the SAS program to perform the statistical analysis, interpretation of the data, and helped to finalize the manuscript.

All authors read and approved the final manuscript.

## Pre-publication history

The pre-publication history for this paper can be accessed here:

http://www.biomedcentral.com/1472-6963/11/103/prepub
